# Motion preservation for open book injuries of the pubic symphysis –a biomechanical cadaver study

**DOI:** 10.1007/s00402-024-05390-7

**Published:** 2024-05-27

**Authors:** Adrian Cavalcanti Kußmaul, Nele Baur, Jan Wulf, Axel Greiner, Rouven Neudeck, Manuel Kistler, Carl Neuerburg, Wolfgang Böcker, Christopher A Becker

**Affiliations:** Department of Orthopaedics and Trauma Surgery, Musculoskeletal University Center Munich (MUM), LMU University Hospital, LMU Munich, Marchioninistr. 15, 81377 Munich, Germany

**Keywords:** Pelvic instability, Biomechanics, Minimally invasive, Flexible osteosynthesis, Pubic symphysis, SpeedBridge™

## Abstract

**Introduction:**

Open book injuries are challenging injuries that oftentimes require surgical treatment. Currently, treatment is performed with symphyseal plating requiring extensive surgery and entirely limiting physiological movement of the symphyseal joint, frequently resulting in implant failure. Therefore, we investigated the biomechanical properties of a minimally invasive tape suture construct (modified SpeedBridge™) as an alternative stabilization technique for the treatment of open book injuries in human cadaver pelvic rings.

**Materials and Methods:**

The symphysis of 9 human cadaver pelvises was dissected and dilated to 3 cm creating an open book injury. Next, the two osteosynthesis methods (plating, modified SpeedBridge™) were applied. All specimens then underwent cyclic horizontal and vertical loading, simulating biomechanical forces while sitting, standing and walking. For statistical analysis, 3D dislocation (mm) was calculated.

**Results:**

Total displacement (mm) of the pubic symphysis displayed the following means and standard deviations: native group 1.34 ± 0.62 mm, open book group 3.01 ± 1.26 mm, tape group 1.94 ± 0.59 mm and plate group 1.37 ± 0.41 mm. Comparison between native and open book (*p* = 0.029), open book and plate (*p* = 0.004), open book and tape (*p* = 0.031), as well as tape and plate group (*p* = 0.002) showed significant differences. No significant differences were found when comparing the native and tape (*p* = 0.059), as well as the native and plate (*p* = 0.999) group.

**Conclusion:**

While both osteosynthesis techniques sufficiently stabilized the injury, symphyseal plating displayed the highest rigidity. The modified SpeedBridge™ as a tape suture construct provided statistically sufficient biomechanical stability while maintaining symphyseal micro mobility, consequently allowing ligamental healing of the injured joint without iatrogenic arthrodesis.

**Supplementary Information:**

The online version contains supplementary material available at 10.1007/s00402-024-05390-7.

## Introduction

When considering pelvic ring injuries, the differentiation between two entities is essential for adequate fracture management: While fragility fractures of the pelvis (FFP) result from low-energy trauma and primarily affect the geriatric population due to compromised bone quality, combined ligamentous / osseous pelvic ring injuries are mostly the result of high-energy trauma. These fractures account for 3-7% of all fractures and are associated with mortality rates of 5-20% [1, 2].

According to the Young and Burgess classification, open book fractures include the rupture of the pubic symphysis and a ligamentous or osseous injury of the posterior pelvic ring as the result of anteroposterior compression (APC type 1 - 3) [3, 4]. Regarding the AO classification, they are classified as AO type 61-B1.2, 61-B2.3, 61-B3.3 or 61-C1-C3 fractures, depending on the involvement of the posterior pelvic ring [5–7]. Yet, the term “fracture” can be considered misleading as this injury oftentimes describes a ligamentous injury without compulsory osseous affection.

Considering the treatment of open book injuries, surgical treatment is oftentimes necessary to restore stability of the pelvic ring. Currently, the gold standard consists of open reduction and internal fixation (ORIF) with symphyseal plating [8]. However, this not only requires extensive and invasive surgery, which is associated with high blood loss, but also bears various perioperative risks such as infection, postoperative bleeding or heterotopic ossification [9, 10]. Another significant complication is implant failure, which can be observed in approximately 61.8 – 80% of the patients [10, 11]. Biomechanically, this can mostly be explained by the rigidity of the plate aiming to nullify the physiological symphyseal micro mobility of approximately 2 mm [9, 12]. This iatrogenic arthrodesis oftentimes fails to neutralize the significant torsion forces acting on the pubic symphysis, ultimately resulting in subsequent implant failure and sometimes even requiring revision surgery [11–13]. To address this problem, various alternative treatment options, such as cerclages, endobutton systems or internal fixators, have been evaluated, yet without significant improvement of failure rates [14–16].

Another promising approach are minimally invasive tape suture constructs (TSC) such as the modified Speedbridge™ (Arthrex, Naples, Florida, USA). These implants allow micro mobility of weight-bearing joints while maintaining sufficient biomechanical stability and are already established for ankle, rotator cuff and knee injuries [17–20]. This technique has already been proven biomechanically equivalently stable to anterior plating for symphyseal disruptions by Cavalcanti Kußmaul et al. for both isolated anterior synthetic and anterior human cadaveric pelvic rings [20, 21]. However, there is no data evaluating the biomechanical properties of TSC for the treatment of symphyseal disruptions in an entire human pelvic ring.

Consequently, this study aims to investigate the biomechanical properties of tape suture constructs for the treatment of open book injuries in an entire human pelvic ring.

## Materials and methods

Prior to the experiments, this biomechanical study was approved by the Ethics Committee of the LMU (approval number: 21 – 0481) and was performed according to the principles of the Helsinki declaration. In this study, 9 fresh frozen human cadaver pelvises were used. The donors written consent was obtained during their lifetime. Prior to preparation, a quantitative computed tomography (qCT) scan was performed for exclusion of any compromising condition such as previous fracture or synostosis.

After thawing the specimens, the surrounding soft tissue was dissected while ligamentous structures were preserved. The lumbar spine was separated between L3 and L4. Then, the specimens were placed with the lumbar spine facing downwards in an individually manufactured aluminum pot and the L4 embedded in resin (RenCast^®^ FC 52/53 Isocyanate/FC 53 Polyol, Huntsman Cooperation^®^, Salt Lake City, UT, USA).

Testing was performed in a single-leg stance mimicking human gait using a dual-head prothesis appropriate to the individual acetabular diameter.

**Fig. 1:**
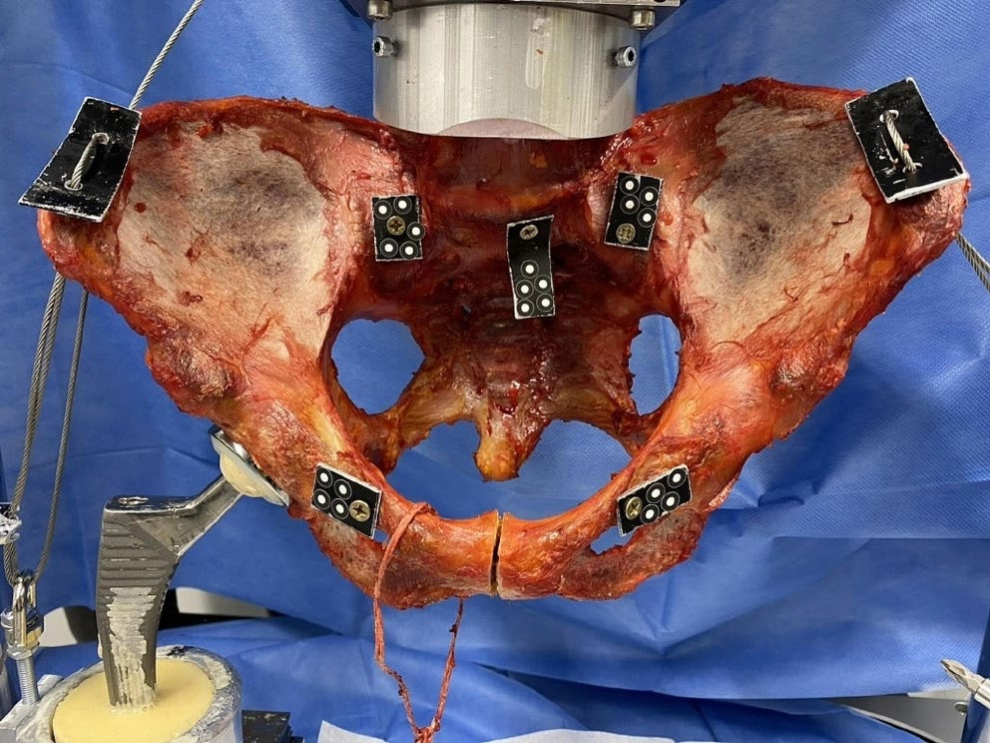
Experimental set-up with a specimen after simulation of an open book injury

**Fig. 2:**
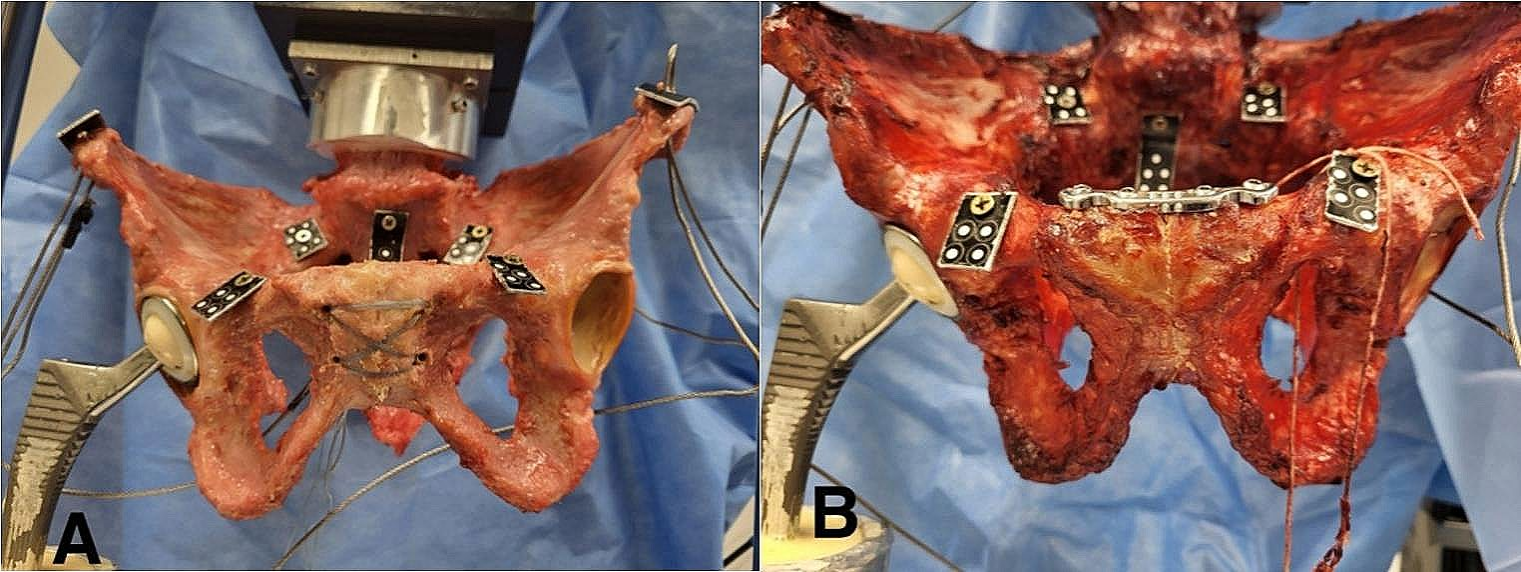
(**a**): Tape suture construct using a modified Speed Bridge™, (**b**): Symphyseal plating

Initially, the intact pelvises were tested forming a reference group (native group) (Fig. 1). Next, the pubic symphysis was transected and expanded to 3 cm using a standardized wedge simulating an open book fracture (open book group). Then, the osteosynthesis techniques were applied: When performing a TSC using a modified Speed Bridge™ (Arthrex, Naples, FL, USA), four holes with two on each side of the symphysis were pre-drilled with a 3.5 mm drill after which the tape was attached using the criss-cross technique (tape group) (Fig. 2a). In the plate group, a symphyseal plate (DePuySynthes 3.5; four holes, dynamic compression plate, Westchester, PA, USA) and four identical cortical screws (DePuySynthes Cortex screw 3.5 mm, 50 mm, Westchester, PA, USA) were placed according to AO surgery reference (Fig. 2b).

**Fig. 3:**
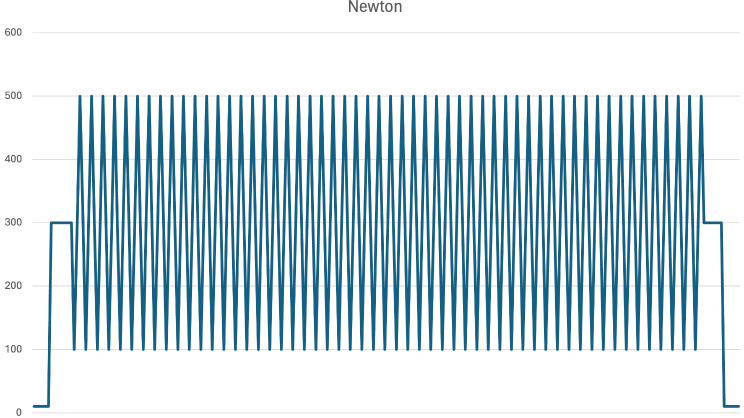
Graphical illustration of the biomechanical loading protocol

Biomechanical loading was performed according to a 5 step protocol (Table 1; Fig. 3). In order to simulate human gait, the specimens were loaded with up to 500 N in the single leg stance set-up corresponding to a 50 kg torso. The force was applied in an axial direction through the lumbar spine and discharged through the dual-head prosthesis.

**Table 1:** Biomechanical loading protocol

Step 1	Pelvic lowered and fitted onto the dual head prosthesis with a axial preload of 10 *N*
Step 2	Compression force loading up to 300 N and holding for 5 s
Step 3	Periodic loading: 55 cycles with a frequency of 1 Hz between 100 and 500 N (+/- 200 N)
Step 4	Back to Compression force loading 300 N and holding for 5 s
Step 5	Decrease loading to initial preload of 10 N

**Table 2:** Descriptive Data

Specimen	Age (years)	Sex
1	93	female
2	74	female
3	73	female
4	80	male
5	79	female
6	94	male
7	77	female
8	85	female
9	96	female

Displacement (mm) of the pubic symphysis was measured during step 3 using an optic sensor system (GOM Aramis 3D Camera 12 M, GOM GmbH, Braunschweig, Germany) for comparison. For this purpose, marker points were created directly next to the pubic symphysis and generated fracture. A measuring distance of around 700 mm and a measuring volume of 560 mm x 380 mm x 380 mm was used for all measurements. The approximate measuring accuracy was 11.2 μm in focus-plane and 22.4 μm out of focus-plane.

### Data analysis and statistics

Data from all 9 specimens were analyzed using Microsoft Excel (Microsoft cooperation; version 16.81). Relative fracture displacement was calculated for every single load cycle (maximum to minimum). This calculation was done in each coordinate axis and afterwards converted into 3D fracture displacement. The resulting displacement and standard deviation (SD) were calculated from 10 cycles for each specimen.

Statistical analysis was performed using GraphPad Prism (version 10.1.0 for macOS, GraphPad Software, Boston, Massachusetts USA). Mean values of all specimens within one group (native, open book, tape, and plate) exhibited a normal distribution when analyzed by Shapiro-Wilk test. Statistical comparison of mean values was achieved using a paired one-way ANOVA with Geisser-Greenhouse correction and Tukey post hoc analysis at a significance level of *p* < 0.05.

## Results

Mean age was 83.4 ± 8.9 years with 7 female and 2 male specimens (Table 2).

**Fig. 4:**
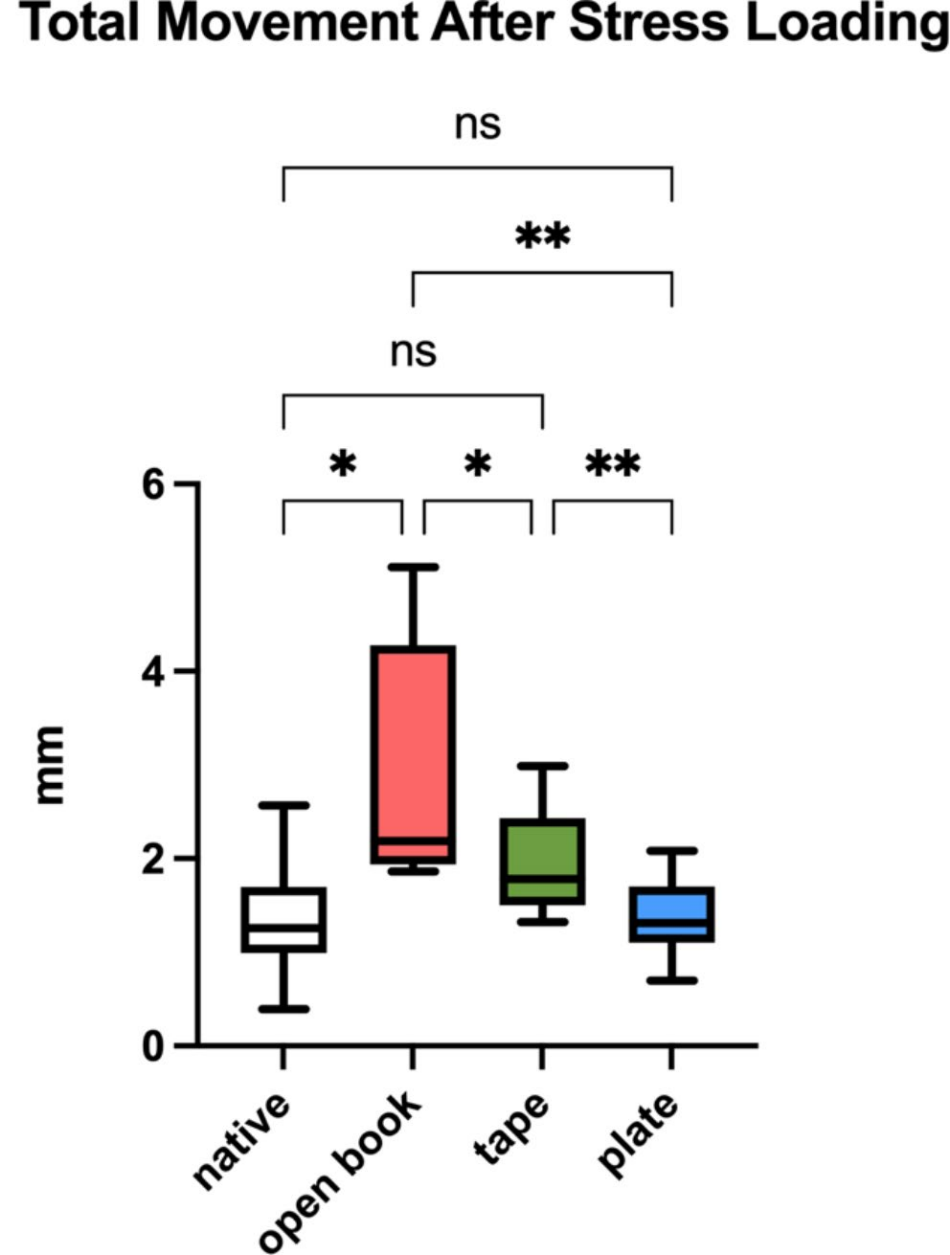
Total symphyseal displacement (mm). Box and whiskers error bars indicate minimum and maximum values of mean symphyseal displacement with non-significant (ns) or significant results at a significance level of *p* < 0.05 (*) or *p* < 0.005 (**). GraphPad Prism (version 10.1.0 for macOS, GraphPad Software, Boston, Massachusetts USA)

Following stress loading, total displacement (mm) of the pubic symphysis displayed the following means and standard deviations: native group 1.34 ± 0.62 mm, open book group 3.01 ± 1.26 mm, tape group 1.94 ± 0.59 mm and plate group 1.37 ± 0.41 mm (Fig. 4). Comparison between native and open book (*p* = 0.029), open book and plate (*p* = 0.004), open book and tape (*p* = 0.031), as well as tape and plate group (*p* = 0.002) showed significant differences (Fig. 4). No significant differences were found when comparing the native and tape (*p* = 0.059), as well as the native and plate (*p* = 0.999) group (Fig. 4).

## Discussion

Pubic symphyseal disruption is oftentimes caused by an anteroposterior compression of the pelvis [5–7]. Surgical treatment is often necessary to restore the stability of the pelvic ring [8]. The current gold standard is open reduction followed by anterior plate osteosynthesis [8]. This surgical procedure amongst others results in an iatrogenic arthrodesis of the symphysis almost nullifying the physiological micromobility of this motion segment, oftentimes leading to implant failure due to screw loosening or breakage [9–13]. Ptunis et al. for example observed this phenomenon in 31% of cases, including 12% resulting in a loss of reduction and 8% needing revision surgery, while Jordan et al. describe radiological failure rates up to 81% [22, 23].

To address this problem, this study investigated the biomechanical properties of a tape suture construct for the treatment of open book injuries in an entire human pelvic ring.

Regarding dislocation in the native condition, this study found a narrow distribution of mean values approaching the physiological values of up to 2 mm found by Walheim et al. in 15 healthy young volunteers, fundamentally reflecting the usability of the data obtained in this study [12].

Also, this study found a 225% higher dislocation between the native (1.34 ± 0.62 mm) and open book group (3.01 ± 1.26 mm) (*p* = 0.029) after stress loading of the pelvis simulating full weight bearing. Fundamentally, these results stress the need for surgical treatment for open book injuries in order to avoid massive instability during loading.

When comparing the native group to both the plate (*p* > 0.999) and the tape group (*p* = 0.059), no significant differences were found, highlighting the sufficient biomechanical stability of both treatment methods. This is also underlined by the comparison of the open book group to both stabilization techniques (open book vs. plate: *p* = 0.004, open book vs. tape: *p* = 0.031).

**Fig. 5:**
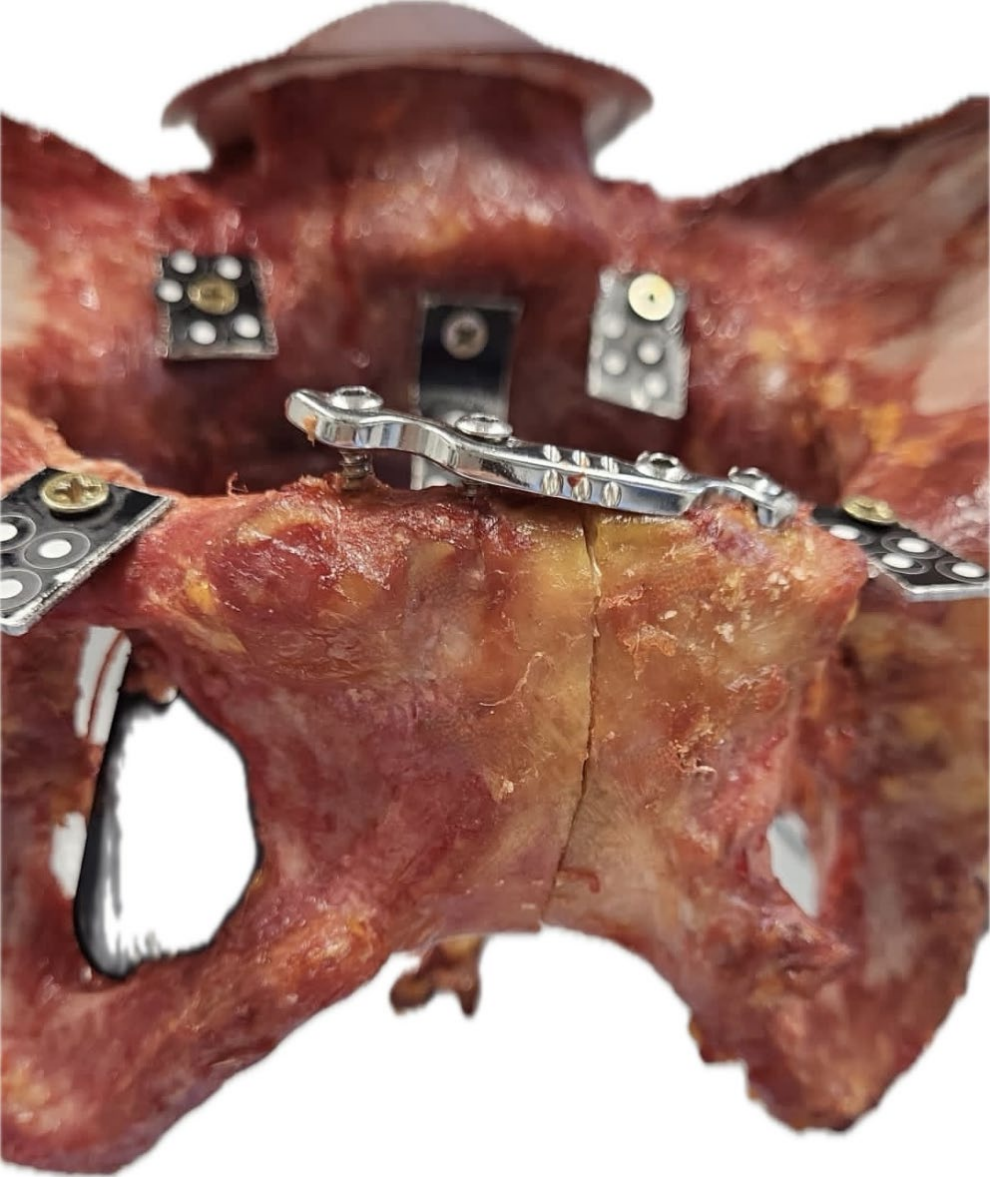
Incipiently screw loosening in anterior plating

In detail, dislocation with anterior plating displays similar mobility to the native condition, showing the incapacity of the plate osteosynthesis to fully withstand the physiological micro mobility of the pubic symphysis and ultimately favoring implant loosening and failure (Fig. 5) [20, 21, 23–25]. Incipiently screw loosening was observed in 2 specimens after cyclic loading, further reflecting the conflict of a rigid osteosynthesis method for the treatment of the symphyseal motion segment (Fig. 5). No anchor dislocation or tape lengthening / rupture occurred in the tape group, highlighting the adequate resistance of TSCs for the treatment of motions segments despite their flexibility.

The significant difference between the plate and tape group (*p* = 0.002) after stress loading further reflects the rigidity of anterior plating and highlights the preservation of symphyseal micro mobility under TSCs.

As another alternative to anterior plating, Jordan et al. investigated the idea of a flexible osteosynthesis for the anterior pelvic ring evaluating the use of a trans-obturator cable in double loop technique. Here, the trans-obturator cable demonstrated similar stability to the plate osteosynthesis. However, this procedure is associated with an increased invasiveness due to the necessity of the preparation of the obturator foramen. Also, based on the need of the cable to pass through the obturator canal, there is a relevant risk of injuring the surrounding vessels and nerves. To minimize this risk, the cable must be placed directly at the medial edge of the obturator foramen. Another possible complication is the horizontal dislocation of the cable with increasing load, which can be reduced by using an anchoring system [26].

As the single use of a cable osteosynthesis might also eventually lead to loosening due to direct bone contact, Jordan et al. investigated the use of a cable-clamp device on both artificial bone and cadaveric pelvises in a further study. In both cases, sufficient biomechanical stability was demonstrated similar to anterior plating [27].

Another approach investigated by Kiskaddon et al. is the use of a suture button construct [28]. This technique is already being used with promising results for syndesmosis injuries of the ankle [29]. Here, the authors were able to demonstrate the feasibility of suture button constructs for the treatment of fractures of the anterior pelvic ring. Yet again, the disadvantage of this technique continues to be the need of dissection around the pubic symphysis, especially increasing the risk of injury to the bladder and the surrounding soft tissue [28].

Previous studies by Cavalcanti Kußmaul et al. also examined the use of a similar TSC in initially synthetic and afterwards in cadaver models with isolated anterior pelvic rings [20, 21]. Here, the testing force for biomechanical loading was lower (75 N in each direction) as the isolated anterior pelvic ring contributes to up to 30% to pelvic stability [21, 30]. However, using an isolated symphysis enables loading in the different motions planes of the symphysis: For tension and vertical shear at the pubic symphysis, the authors found a maximum dislocation of 0.30 ± 0.12 mm and 0.59 ± 0.20 respectively. These very small dislocations of below 1 mm are likely due to the loading forces [21]. Also, the pelvic ring acts as a coherent structure and should be considered a unit, which is why in this study a complete pelvic ring was used. Applying the TSC and plate osteosynthesis on the pelvic ring and using a loading force of up to 500 N approaches physiological conditions mimicking human gait. Regarding the loosening of two screws in one specimen in the plate osteosynthesis group (Fig. 5) after only 55 cycles, it can be assumed that the loading forces used in this study were adequate.

Regarding the limitations of this study, the specimens were tested in a single-leg stance resulting in a limited transferability of the obtained results to physiological human stance. Also, the muscles of the pelvis have a major impact on the loading capacity of the pelvis: During normal human gait, the line of gravity shifts in front of or behind the pivot point of the hip depending on the stance and swing leg phase. This change is accommodated by the muscles of the hip, allowing physiological gait [31]. Yet with cadaveric testing, the soft tissue function is limited, restricting transferability of results to in vivo conditions. Furthermore, loading to failure was not performed in this study, thus it remains unclear how the TSC performs under higher loading conditions or whether the failure load of the TSC is comparable to the one of the plate osteosynthesis. Therefore, further clinical and biomechanical studies are necessary to fully comprehend the benefits of TSCs.

## Conclusions

The TSC provides sufficient biomechanical stability for the treatment of symphyseal instabilities and disruptions in a cadaver model while maintaining micro mobility of the pubic symphysis, ultimately avoiding an unphysiological iatrogenic arthrodesis.

Therefore, the TSC could potentially function as an alternative to symphyseal plating for ligamentous open book injuries of the pelvic ring.

### Electronic supplementary material

Below is the link to the electronic supplementary material.

Supplementary Material 1

Supplementary Material 2

Supplementary Material 3

Supplementary Material 4

Supplementary Material 5

Supplementary Material 6

Supplementary Material 7

Supplementary Material 8

Supplementary Material 9
